# Supplementary material for: Targeting the B1 Gene and Analysis of Its Polymorphism Associated with Awned/Awnless Trait in Russian Germplasm Collections of Common Wheat
Source: Plants (Basel). 2021 Oct 25;10(11):2285. doi: 10.3390/plants10112285 (PMC8621087; doi:10.3390/plants10112285)
Supplement: Supplementary file 1 [file plants-10-02285-s001.zip › Table S2.pdf]

Table S2. Phenotyping and genotyping by markers to B1/b1 alleles of winter wheat accessions of Russian wheat germplasm collection of VIR

n\n	VIR catalog number	Sample name	*Subspecies	Origin	PCR test dominant allele (B1for/Znfrev) - recessive allele (b1for/Znfrev)	phenotype, Orel region
1	4784	Banatka	****erythrospermum**	Russia, Stavropol region	***b1***	**awned**
2	4832	-	**erythrospermum**	Russia, Kalmykia	***b1mite***	**awned**
3	9723	Beloturka bezostaya	albidum	Russia, Yaroslavl' region	*B1*	awnless
4	9765	Local	milturum	Russia, Pskov region	*B1*	awnless
5	9789	Batishchevskaya	lutescens	Russia, Smolensk region	*B1*	awnless
6	10213	Gorkonkur	**ferrugineum**	Russia, Kursk region	***b1mite***	**awned**
7	10376	Kosobryukhovka	-	Russia, Krasnodar region	***b1mite***	**awned**
8	10890	Donka	**ferrugineum**	Russia, Rostov region	***b1mite***	**awned**
9	10896	Veshkinskaya	**ferrugineum**	Russia, Moscow region	***b1mite***	**awned**
10	13092	-	milturum	Russia, Primorsky krai	*B1*	awnless
11	22418	Sandomirka	albidum	Russia, Orel region	*B1*	.awnless
12	24500	Mikhaylovka	**erythrospermum**	Russia, Leningrad region	***b1***	**awned**
13	33053	-	**erythrospermum**	Russia, Khabarovsky krai	***b1mite***	**awned**
14	34886	Staritsinskaya	**erythrospermum**	Russia, Tomsk region	***b1***	**awned**
15	35698	Batetskaya belokoloska	albidum	Russia, Novgorod region	*B1*	awnless
16	35757	Pashinskaya	milturum	Russia, Kirov region	*B1*	awnless
17	35766	Bogorodskaya	**ferrugineum**	Russia, Nizhniy Novgorod region	***b1mite***	**awned**
18	36454	Plyusskaya	albidum, lutescens, milturum	Russia, Pskov region	*B1*	awnless
19	36507	Mestnaya	lutescens	Russia, Chuvashia	*B1*	awnless
20	36526	Eritrospermum 59	**erythrospermum**	Russia, Kursk region	***b1mite***	**awned**
21	36590	Skala	lutescens	Russia, Irkutsk region	*B1*	awnless
22	36612	Velizhanskaya	milturum	Russia, Tyumen' region	*B1*	awnless
23	37478	Shatilovskaya	**erythrospermum**	Russia, Altai region	***b1mite***	**awned**
24	38248	Ust'-Ishimka	lutescens	Russia, Omsk region	*B1*	awnless
25	38321	Ustyuzhenskaya	albidum	Russia, Vologda region	*B1*	awnless
26	38342	Mestnaya maslyaninskaya	**erythrospermum**	Russia, Novosibirsk region	***b1mite***	**awned**
27	38435	Sumarokovskaya mestnaya	milturum	Russia, Kostroma region	*B1*	awnless
28	38438	Oyashinskaya	**ferrugineum**	Russia, Novosibirsk region	***b1mite***	**awned**
29	40579	Karel'skaya bezostaya	velutinum	Russia, Karelia	*B1*	awnless
30	42790	Bezostaya 1	lutescens	Russia, Krasnodar region	*B1*	awnless
31	43920	Mironovskaya 808	lutescens	USSR, Ukrainian SSR	*B1*	awnless
32	62733	Inna	lutescens	Russia, Moscow region	*B1*	awnless
33	45654	Kavkaz	lutescens	Russia, Krasnodar region	*B1*	awnless
34	49271	Arthur 71	lutescens	USA	*B1*	awnless
35	62735	Don 93	lutescens	Russia, Rostov region	*B1*	awnless
36	51829	Oasis	lutescens	USA	*B1*	awnless
37	54646	Donskaya bezostaya	lutescens	Russia, Rostov region	*B1*	awnless
38	55798	Ul'yanovka 9	lutescens	Russia, Kursk region	*B1*	awnless
39	55801	Lyutestsens 12	lutescens	Russia, Kursk region	*B1*	awnless
40	56058	Sibiryachka	lutescens	Russia, Novosibirsk region	*B1*	awnless
41	57687	Voronezhskaya 174	lutescens	Russia, Voronezh region	*B1*	awnless

42	58137	Kosack	lutescens	Sweden	*B1*	awnless
43	58676	Stavropol'skaya kormovaya	lutescens	Russia, Stavropol region	*B1*	awnless
44	58802	Labinka	**erythrospermum**	Russia, Krasnodar region	***b1***	**awned**
45	60705	Prikumskaya 986	lutescens	Russia, Stavropol region	*B1*	awnless
46	61531	Omskaya ozimaya	lutescens	Russia, Omsk region	*B1*	awnless
47	61548	Sibirskaya Niva	lutescens	Russia, Omsk region	*B1*	awnless
48	61610	TAM 200	**erythrospermum**	USA	***b1***	**awned**
49	61966	Bezenchukskaya 380	lutescens	Russia, Samara region	*B1*	awnless
50	61992	Eritrospermum 31	**erythrospermum**	Russia, Orenburg region	***b1***	**awned**
51	62019	Fundulea 4	**erythrospermum**	Romania	***b1***	**awned**
52	62372	Karl	**erythrospermum**	USA	***b1***	**awned**
53	62399	Century	**erythrospermum**	USA	***b1***	**awned**
54	62432	Kazanskaya 285	**erythrospermum**	Russia, Tatarstan	***b1***	**awned**
55	62452	Thunderbird	**erythrospermum**	USA	***b1***	**awned**
56	62509	Tundza	lutescens	Bulgaria	*B1*	awnless
57	62703	Norkan	**erythrospermum**	USA	***b1***	**awned**
58	62707	Abilene	**erythrospermum**	USA	***b1***	**awned**
59	62711	Victory	**erythrospermum**	USA	***b1***	**awned**
60	44132	Ramonskaya 42	**ferrugineum**	Russia, Voronezh region	***b1***	**awned**
61	49880	Voronezhskaya 34	**hostianum**	Russia, Voronezh region	***b1***	**awned**
62	62738	Zernogradka 9	lutescens	Russia, Rostov region	*B1*	awnless
63	62744	Peacock	lutescens	Great Britain	*B1*	awnless
64	63001	Suzdal'skaya 2	lutescens	Russia, Vladimir region	*B1*	awnless
65	63002	Tau	lutescens	Russia, Vladimir region	*B1*	awnless
66	63006	Tjelvar	lutescens	Sweden	*B1*	awnless
67	63037	Tarasovskaya ostistaya	**erythrospermum**	Russia, Rostov region	***b1***	**awned**
68	63040	Zimorodok	lutescens	Russia, Krasnodar region	*B1*	awnless
69	63104	Orenburgskaya 12	velutinum	Russia, Orenburg region	*B1*	awnless
70	63106	Guberniya	lutescens	Russia, Saratov region	*B1*	awnless
71	63117	Splav	lutescens	Russia, Vladimir region	*B1*	awnless
72	63119	Zalarinka	lutescens	Russia, Irkutsk region	*B1*	awnless
73	63368	Yasen	**erythrospermum**	Bulgaria	***b1***	**awned**
74	63523	Vista	**erythrospermum**	USA	***b1***	**awned**
75	63565	Kazanskaya 560	**erythrospermum**	Russia, Tatarstan	***b1***	**awned**
76	63894	Mona	lutescens	Czech Republic	*B1*	awnless
77	63897	Encore	lutescens	Great Britain	*B1*	awnless
78	63905	Bruden=Bryden	lutescens	Great Britain	*B1*	awnless
79	63922	Zodiac	lutescens	Great Britain	*B1*	.awnless
80	63981	Turda 81-77	lutescens	Romania	*B1*	awnless
81	64008	Gorbi	lutescens	Germany	*B1*	awnless
82	64024	Carolus	lutescens	Germany	*B1*	awnless
83	64033	Arber	lutescens	Germany	*B1*	awnless
84	64049	Rawhide	**erythrospermum**	USA	***b1***	**awned**
85	64058	Zlatostrui	**graecum**	Bulgaria	***b1***	**awned**
86	64066	Vida	lutescens	Bulgaria	*B1*	awnless
87	64160	Moskovskaya 39	**erythrospermum**	Russia, Moscow region	***b1***	**awned**
88	64161	Galina	**erythrospermum**	Russia, Moscow region	***b1***	**awned**
89	64182	Mv.Madrigal	lutescens	Hungary	*B1*	awnless

90	64183	Mv.Magdalena	**erythrospermum**	Hungary	***b1***	**awned**
91	64184	Mv.Szigma	lutescens	Hungary	*B1*	awnless
92	64186	Rapid	**erythrospermum**	Romania	***b1***	**awned**
93	64187	Dropia	**erythrospermum**	Romania	***b1***	**awned**
94	64188	Ariesan	**ferrugineum**	Romania	***b1***	**awned**
95	64192	Alex	**erythrospermum**	Romania	***b1***	**awned**
96	64198	Mera	lutescens	Russia, Vladimir region	*B1*	.awnless
97	64280	Biryuza	lutescens	Russia, Samara region	*B1*	awnless
98	64350	Bat'ko	lutescens	Russia, Krasnodar region	*B1*	awnless
99	64521	Autan	**erythrospermum**	France	***b1***	**awned**
100	64522	Brando	lutescens	France	*B1*	awnless
101	64523	Caphorn	lutescens	France	*B1*	.awnless
102	64525	Forban	lutescence	France	*B1*	awnless
103	64526	Hamac	lutescens	France	*B1*	.awnless
104	64527	Isengrain	**erythrospermum**	France	***b1***	**awned**
105	64535	Vulcain	lutescens	France	*B1*	awnless
106	64620	Dominanta	**erythrospermum**	Russia, Rostov region	***b1***	**awned**
107	64632	Volzhskaya S3	**erythrospermum**	Russia, Ul'yanovsk region	***b1***	**awned**
108	64747	Novosibirskaya 51	lutescens	Russia, Novosibirsk region	*B1*	awnless
109	64749	Novosibirskaya 9	lutescens	Russia, Novosibirsk region	*B1*	awnless
110	64909	Zhemchuzhina Povolzh'ya	lutescens	Russia, Saratov region	*B1*	awnless
111	64934	Superior	albidum	Canada	*B1*	awnless
112	64936	AC Tempest	**ferrugineum**	Canada	***b1***	**awned**
113	65027	Karat	lutescens	Bulgaria	*B1*	awnless
114	65035	Mv.Koma	**graecum**	Hungary	***NA***	**awned**
115	65068	Siluet	**compositumferrugineum**	Russia, Volgograd region	***NA***	**awned**
116	65069	Status	compositummilturum	Russia, Volgograd region	*B1*	awnless
117	65174	Webster	lutescens	Canada	*B1*	awnless
118	65175	Wisdom	lutescens	Canada	*B1*	awnless
119	65192	L'govskaya 4	lutescens	Russia, Kursk region	*B1*	awnless
120	65207	Ermak	**erythrospermum**	Russia, Rostov region	***b1***	**awned**
121	65216	Berezit	lutescens	Russia, Stavropol region	*B1*	awnless
122	65217	Kseniay	**erythrospermum**	Russia, Stavropol region	***b1***	**awned**
123	65233	Donstar	lutescens	Russia, Rostov region	*B1*	awnless
124	65292	GK Margit	lutescens	Hungary	*B1*	awnless
125	65331	FT Wonder	**erythrospermum**	Canada	***b1***	**awned**
126	65375	Don 105	lutescens	Russia, Rostov region	*B1*	awnless
127	65397	Intrada	**graecum**	USA	***b1***	**awned**
128	65405	Amigo	**erythrospermum**	USA	***b1***	**awned**
129	65406	Regina	lutescens	Czech Republic	*B1*	awnless
130	65407	Senta	lutescens	Czech Republic	*B1*	awnless
131	65408	Zdar	lutescens	Czech Republic	*B1*	awnless
132	65409	Boka	lutescens	Czech Republic	*B1*	awnless
133	65610	Dzhangal	albidum	Russia, Saratov region	*B1*	awnless
134	65613	Kalach 60	lutescens	Russia, Saratov region	*B1*	awnless
135	65619	Sakwa	lutescens	Poland	*B1*	awnless
136	65632	Nutka	lutescens	Poland	*B1*	awnless
137	65635	Turnia	lutescens	Poland	*B1*	awnless

138	65638	Certo	lutescens	Germany	*B1*	awnless
139	65672	Firuza 40	**erythrospermum**	Russia, Stavropol region	***b1***	**awned**
140	65756	Nemchinovskaya 17	**erythrospermum**	Russia, Moscow region	***b1***	**awned**
141	65923	Bona Dea	lutescens	Slovakia	*B1*	awnless
142	65927	Malvina	lutescens	Slovakia	*B1*	.awnless
143	65928	Malyska	lutescens	Slovakia	*B1*	awnless
144	65929	Markola	lutescens	Slovakia	*B1*	awnless
145	65935	Verita	lutescens	Slovakia	*B1*	awnless
146	66053	Kristy	lutescens	Canada	*B1*	awnless
147	66054	AC Buteo	**erythrospermum**	Canada	***b1***	**awned**
148	66306	Kamyshanka 4	lutescens	Russia, Volgograd region	*B1*	awnless
149	66308	Torrild	lutescens	Germany	*B1*	awnless
150	66309	Omskaya 5	lutescens	Russia, Omsk region	*B1*	awnless
151	66321	Kobra	lutescens	Poland	*B1*	awnless
152	66322	Soraja	lutescens	Poland	*B1*	awnless
153	66323	Bogatka	lutescens	Poland	*B1*	awnless
154	66325	Muza	lutescens	Poland	*B1*	awnless
155	66332	Lidiya	**erythrospermum**	Russia, Rostov region	***b1***	**awned**
156	66336	Kolos Orenbuzhiya	lutescens	Russia, Orenburg region	*B1*	awnless
157	66337	Pionerskaya 32	**erythrospermum**	Russia, Orenburg region	***b1***	**awned**
158	66341	Bazalt 2	lutescens	Russia, Voronezh region	*B1*	awnless
159	66504	Bazis	**erythrospermum**	Russia, Samara region	***b1***	**awned**
160	66626	Priirtyshskaya	lutescens	Russia, Omsk region	*B1*	awnless
161	66628	Elanskaya	**erythrospermum**	Russia, Volgograd region	***b1***	**awned**
162	66805	Bezostaya 100	lutescens	Russia, Krasnodar region	*B1*	awnless
163	66816	Caravan	lutescens	Russia, Krasnodar region	*B1*	awnless
164	66827	Hamlet	lutescens	Germany	*B1*	awnless
165	66842	Hamdan	lutescens	Russia, Kalmykia	*B1*	awnless
166	66843	Sarul	lutescens	Russia, Kalmykia	*B1*	awnless
167	66883	Riphey	**erythrospermum**	Russia, Orenburg region	***b1***	**awned**
168	67211	Darina	**erythrospermum**	Russia, Tatarstan	***b1***	**awned**
169	67213	Nadezhda	lutescens	Russia, Tatarstan	*B1*	awnless

* Zuev E.V., Amri A., Brykova A.N., Pyukkenen V.P., Mitrofanova O.P. Atlas of bread wheat (*Triticum aestivum L.*) genetic diversity based on spike and kernel characters. Supervising editor N.P. Goncharov. VIR, ICARDA. – 2nd edition. St. Petersburg, 2019. 132 p.

** awned varieties are highlighted in bold
