# Supplementary material for: Targeting the B1 Gene and Analysis of Its Polymorphism Associated with Awned/Awnless Trait in Russian Germplasm Collections of Common Wheat
Source: Plants (Basel). 2021 Oct 25;10(11):2285. doi: 10.3390/plants10112285 (PMC8621087; doi:10.3390/plants10112285)
Supplement: Supplementary file 1 [file plants-10-02285-s001.zip › TableS1.pdf]

Table S1. Specific primers for PCR- amplification of different regions of *C2H2Zf* gene.

Primer pair №	Gene region	Purpose	Forward and reverse primer sequence (5′→3′)	T annealing, °C	PCR program	Size of PCR product, bp
1	Coding	Sequencing	Zfcodf: GTTGTGTTGTGACAAGGGCTG	58	Touch-down 1	438
			Zfcodr: CTACTGCATGTCCTTAGTGCCT	58		
2	Promoter	Sequencing	Zfpromf1: GACGGGAAGGCACTTAAACC	56	Touch-down 1	1264/1097**
			Zfpromf2* GGAGGATGAACTTGAGCTAGGGG Zfpromr: ATCGAGCCCCTCTTCCATCTCC	60		
			Zfpromf3*: GGTTGGTCATATCAACCATGAC	55		
3	Promoter	Targeting (*B1*- allele)	B1for: ATAAACTCCCACATAATTACTTCG	52	Touch-down 2	1177
			Znfrev: CTCTTCCATCTCCATGCCCA	57		
4	Promoter	Targeting (*b1*-allele)	b1for: AAACTCCCACATAATTACTCCC	53	Touch-down 2	1175
			Znfrev: CTCTTCCATCTCCATGCCCA	57		

*- Additional primers for sequencing; **-PCR product in case of combination Zfpromf2/Zfpromr
