# Supplementary material for: Targeting the B1 Gene and Analysis of Its Polymorphism Associated with Awned/Awnless Trait in Russian Germplasm Collections of Common Wheat
Source: Plants (Basel). 2021 Oct 25;10(11):2285. doi: 10.3390/plants10112285 (PMC8621087; doi:10.3390/plants10112285)
Supplement: Supplementary file 1 [file plants-10-02285-s001.zip › o┤able S3.pdf]

**Table S3** Determined *B1*- genotypes of awnless plants of *T. aestivum* from the F2 population obtained by crossing Lubava 5 (awned) x 476-10 line (awnless). 1039-1062- numbers indicate F1 plants. Homozygous *B1B1* genotypes are in bold.

| **Numbers of F2 plants** | **PCR results** B1for/Znfrev // b1for/Znfrev | **Genotype** | **F3 population, segregation into awned and awnless forms** |
|---|---|---|---|
| 1039-1 | 1177 // 1175 | B1b1 | present |
| 1039-2 | 1177 // 1175 | B1b1 | present |
| 1039-3 | 1177 // 1175 | B1b1 | present |
| 1039-4 | 1177 // 1175 | B1b1 | present |
| 1039-5 | 1177 // 0 | **B1B1** | **absent** |
| 1039-6 | 1177 // 0 | **B1B1** | **absent** |
| 1039-7 | 1177 // 1175 | B1b1 | present |
| 1039-8 | 1177 // 1175 | B1b1 | present |
| 1039-9 | 1177 // 1175 | B1b1 | present |
| 1039-10 | 1177 // 1175 | B1b1 | present |
| 1039-11 | 1177 // 1175 | B1b1 | present |
| 1039-12 | 1177 // 1175 | B1b1 | present |
| 1039-13 | 1177 // 1175 | B1b1 | present |
| 1039-14 | 1177 // 1175 | B1b1 | present |
| 1039-15 | 1177 // 1175 | B1b1 | present |
| 1039-16 | 1177 // 1175 | B1b1 | present |
| 1044-1 | 1177 // 1175 | B1b1 | present |
| 1044-2 | 1177 // 1175 | B1b1 | present |
| 1044-3 | 1177 // 1175 | B1b1 | present |
| 1044-4 | 1177 // 0 | **B1B1** | **absent** |
| 1044-5 | 1177 // 1175 | B1b1 | present |
| 1044-6 | 1177 // 0 | **B1B1** | **absent** |
| 1044-7 | 1177 // 1175 | B1b1 | present |
| 1044-8 | 1177 // 1175 | B1b1 | present |
| 1044-9 | 1177 // 0 | **B1B1** | **absent** |
| 1044-10 | 1177 // 0 | **B1B1** | **absent** |
| 1044-11 | 1177 // 1175 | B1b1 | present |
| 1044-12 | 1177 // 1175 | B1b1 | present |
| 1049-1 | 1177 // 0 | **B1B1** | **absent** |
| 1049-2 | 1177 // 0 | **B1B1** | **absent** |
| 1049-3 | 1177 // 0 | **B1B1** | **absent** |
| 1049-4 | 1177 // 0 | **B1B1** | **absent** |
| 1049-5 | 1177 // 1175 | B1b1 | present |
| 1049-6 | 1177 // 0 | **B1B1** | **absent** |
| 1049-7 | 1177 // 0 | **B1B1** | **absent** |
| 1049-8 | 1177 // 1175 | B1b1 | present |
| 1049-9 | 1177 // 1175 | B1b1 | present |
| 1049-10 | 1177 // 1175 | B1b1 | present |
| 1049-11 | 1177 // 1175 | B1b1 | present |
| 1049-12 | 1177 // 1175 | B1b1 | present |
| 1054-1 | 1177 // 0 | **B1B1** | **absent** |
| 1054-2 | 1177 // 1175 | B1b1 | present |
| 1054-3 | 1177 // 1175 | B1b1 | present |
| 1054-4 | 1177 // 1175 | B1b1 | present |
| 1054-5 | 1177 // 1175 | B1b1 | present |

| | | | |
|---|---|---|---|
| 1054-6 | 1177 // 1175 | B1b1 | present |
| 1054-7 | 1177 // 1175 | B1b1 | present |
| 1054-8 | 1177 // 1175 | B1b1 | present |
| 1054-9 | 1177 // 1175 | B1b1 | present |
| 1054-10 | 1177 // 1175 | B1b1 | present |
| 1054-11 | 1177 // 0 | **B1B1** | **absent** |
| 1054-12 | 1177 // 1175 | B1b1 | present |
| 1054-13 | 1177 // 0 | **B1B1** | **abcent** |
| 1054-14 | 1177 // 1175 | B1b1 | present |
| 1055-1 | 1177 // 0 | **B1B1** | **absent** |
| 1055-2 | 1177 // 1175 | B1b1 | present |
| 1055-3 | 1177 // 1175 | B1b1 | present |
| 1055-4 | 1177 // 1175 | B1b1 | present |
| 1055-5 | 1177 // 0 | **B1B1** | **absent** |
| 1055-6 | 1177 // 1175 | B1b1 | present |
| 1055-7 | 1177 // 1175 | B1b1 | present |
| 1055-8 | 1177 // 0 | **B1B1** | **absent** |
| 1055-9 | 1177 // 1175 | B1b1 | present |
| 1055-10 | 1177 // 0 | **B1B1** | **absent** |
| 1056-1 | 1177 // 1175 | B1b1 | present |
| 1056-2 | 1177 // 0 | **B1B1** | **absent** |
| 1056-3 | 1177 // 1175 | B1b1 | present |
| 1056-4 | 1177 // 1175 | B1b1 | present |
| 1056-5 | 1177 // 0 | **B1B1** | **absent** |
| 1056-6 | 1177 // 1175 | B1b1 | present |
| 1056-7 | 1177 // 1175 | B1b1 | present |
| 1056-8 | 1177 // 0 | **B1B1** | **absent** |
| 1056-9 | 1177 // 1175 | B1b1 | present |
| 1056-10 | 1177 // 1175 | B1b1 | present |
| 1062-1 | 1177 // 1175 | B1b1 | present |
| 1062-2 | 1177 // 1175 | B1b1 | present |
| 1062-3 | 1177 // 1175 | B1b1 | present |
| 1062-4 | 1177 // 0 | **B1B1** | **absent** |
| 1062-5 | 1177 // 0 | **B1B1** | **absent** |
| 1062-6 | 1177 // 1175 | B1b1 | present |
| 1062-7 | 1177 // 1175 | B1b1 | present |
| 1062-8 | 1177 // 1175 | B1b1 | present |
| 1062-9 | 1177 // 1175 | B1b1 | present |
| 1062-10 | 1177 // 1175 | B1b1 | present |
| 1062-11 | 1177 // 1175 | B1b1 | present |
| 1062-12 | 1177 // 1175 | B1b1 | present |
| 1062-13 | 1177 // 0 | **B1B1** | **absent** |
| 1062-14 | 1177 // 0 | **B1B1** | **absent** |
| 1062-15 | 1177 // 1175 | B1b1 | present |
| 1062-16 | 1177 // 0 | **B1B1** | **absent** |
| 1062-17 | 1177 // 1175 | B1b1 | present |
