# Supplementary figures and images for: Targeting the B1 Gene and Analysis of Its Polymorphism Associated with Awned/Awnless Trait in Russian Germplasm Collections of Common Wheat
Source: Plants (Basel). 2021 Oct 25;10(11):2285. doi: 10.3390/plants10112285 (PMC8621087; doi:10.3390/plants10112285)

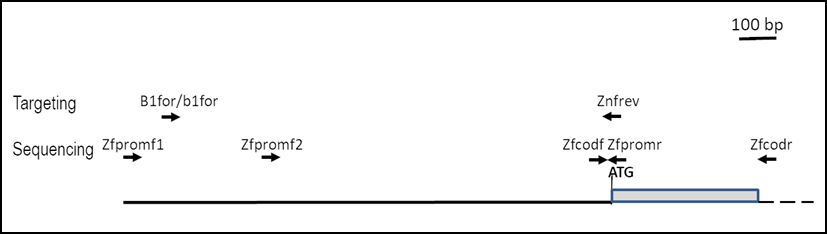

Supplement: Supplementary file 1 [file plants-10-02285-s001.zip › FigureS1.tif]

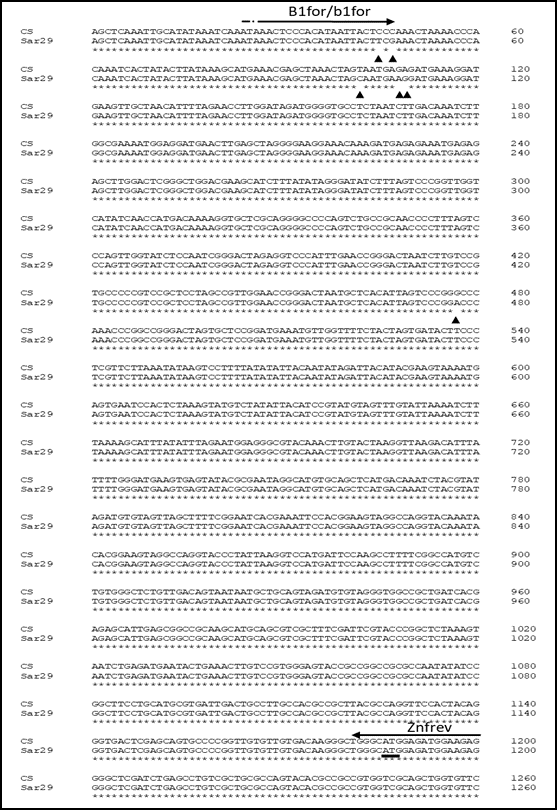

Supplement: Supplementary file 1 [file plants-10-02285-s001.zip › FigureS2.tif]

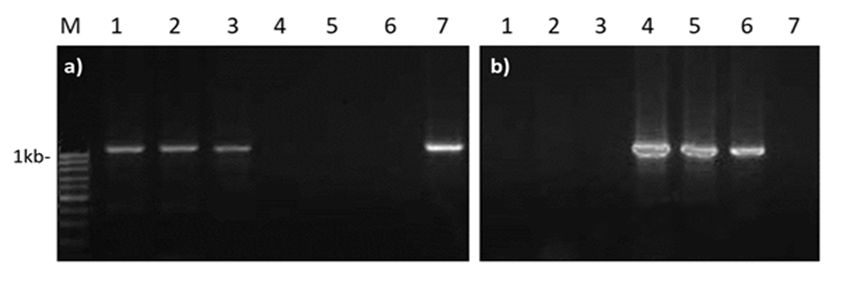

Supplement: Supplementary file 1 [file plants-10-02285-s001.zip › FigureS3.tif]

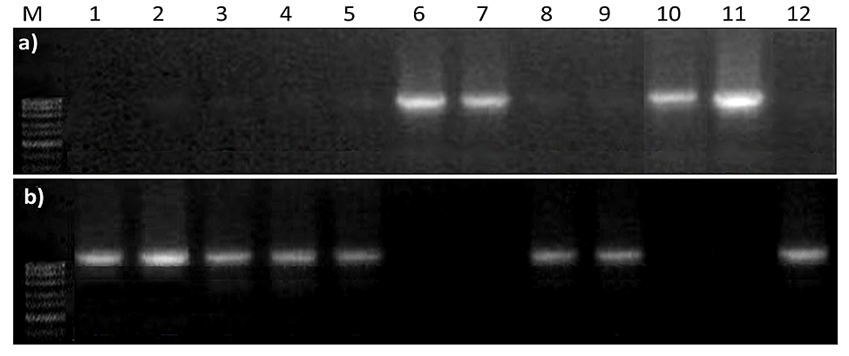

Supplement: Supplementary file 1 [file plants-10-02285-s001.zip › FigureS4.tif]
